# Testis Cancer: Genes, Environment, Hormones

**DOI:** 10.3389/fendo.2014.00172

**Published:** 2014-10-21

**Authors:** Alberto Ferlin, Carlo Foresta

**Affiliations:** ^1^Centre for Human Reproduction Pathology, Department of Medicine, University of Padova, Padova, Italy

**Keywords:** testis cancer, genetics, germ cell tumors, KITLG

Testicular cancer (TC) is the most common cancer in males aged 20–40 years, with a worldwide incidence of 7.5 per 100,000, but the rates vary considerably between countries and ethnic groups and there is evidence also for an increasing incidence in last decades. About 95% of all TCs are represented by testicular germ cell tumors (TGCTs), which include seminoma and non-seminoma histological types. It is generally assumed that the development of TGCT is under endocrine control. In particular, unbalanced androgen/estrogen levels and/or activity are believed to represent the key events for TGCT development and progression.

Furthermore, recent evidence has suggested genetic association of TGCT with variations in genes involved in hypothalamic–pituitary–testicular axis and steroidogenic enzymes (1). This recent evidence expands the current knowledge on the role of genetic contribution in TC susceptibility, and supports the hypothesis that variations in hormone metabolism genes might change the hormonal environment implicated in testicular carcinogenesis. Therefore, hormonal carcinogenesis is an important and controversial area of current research in TGCT, and further attention is given to genetic factors influencing hormone-related cancer risk. The genetic component to TGCT is in general strong. In fact, although environmental factors clearly contribute to TGCT development (and probably to its increasing incidence in some geographical areas), the proportion of TGCT susceptibility accounted for by the genetic effects is estimated at 25%. TGCT has high familial risks compared with most other cancer types that are generally not more than twofold: brothers of individuals with TGCT have an 8- to 12-fold increased risk of disease, and sons of affected individuals have a 4- to 6-fold increased risk (2). Despite this strong familial relative risk, early results from linkage studies identified a limited relationship with genetic factors, suggesting that TGCT is a genetically complex trait. However, more recently, genome-wide association studies (GWAS) have reported association of TGCTs with new loci (such as those in KITLG, SPRY4, BAK1, DMRT1, TERT, and ATF7IP genes). The strongest association for TGCT susceptibility was found for SNPs in KITLG (ligand for the membrane-bound receptor tyrosine kinase KIT) gene with a >2.5-fold increased risk of disease per major allele, which is the highest reported for any cancer to date (3, 4). These studies are being now replicated (5) and attention is given to the relationship between these genetic variations, TGCT risk, and frequently associated anomalies of the reproductive tract, such as cryptorchidism and infertility (6). Finally, over the past few decades, TCGT research has focused also on external environmental causes acting mainly as endocrine disrupters of androgen and estrogen pathways, even during the fetal development of the testis (1). It is well known that the testicular dysgenesis syndrome (TDS) hypothesis, proposed 10 years ago, suggests that disturbed testicular development in fetal life may result in one or more of four disorders postnatally, named cryptorchidism, hypospadias, poor semen quality, and TGCT (7). These four disorders are therefore considered as one clinical entity and are linked together by epidemiological and pathophysiological relations. The relative contribution of genetics and environment in TGCT development, and the interactions between endocrine disruptors and variations in genes involved in hormonal carcinogenesis is therefore another interesting area of research.

The aim of this Research Topic is to give an update on the most controversial issues in research areas of TGCT, as well as original contributions giving novel hypotheses and understandings on the pathogenesis and progression of TGCT. Both clinical and basic researches are reported and many questions are addressed. One of these deals with analysis of anthropometric measures and risk of TGCT (8), an obscure matter that this study clarified, showing that TC is positively associated with height and negatively associated with body mass index, indirectly suggesting that modifications in hormonal factors and food intake during childhood and puberty might influence susceptibility to cancer. The effects of endocrine disruptors during *in utero* and/or neonatal development have been a matter of discussion and areas of uncertainty exists. One of these is the possible epigenetic modification following exposure to these molecules and Vega and colleagues review this topic showing how chromatin modifications, which can affect testicular physiology might increase susceptibility to TGCT and the potential molecular pathways involved in these alterations in the context of environmental exposures (9).

Not only TGCT development and progression but also treatment outcome and risks of complications from cisplatin chemotherapy seem to be genetically determined. This is well documented by Fung and colleagues, who showed for the first time that a polymorphism in *ARVCF* gene influences TGCT outcome, carriers of risk allele being exposed to a higher risk of refractory TGCT after initial chemotherapy (10). Furthermore, patients who received chemotherapy had a higher risk of developing semen HPV infection (11), potentially exposing these subjects to HPV-related disorders and worse fertility prognosis.

Physiological and genetic aspects of primordial germ cells, gonocytes, and spermatogenic stem cells transformation are also interesting research areas. One report reviewed the current knowledge on the biology of the postnatal germ cell development (12), another one looked for possible modifications, caused by copy number variations, in genes involved in cell migration of primordial germ cells (13), and Brokken and colleagues showed that polymorphisms in the aryl hydrocarbon receptor repressor gene (that modulates the effects of environmental pollutants) are associated with progression of TGCT to invasive forms (14).

Finally, this Research Topic reports review on the association between cryptorchidism and TGCT (15, 16), clarifying that, regardless of age at orchidopexy, unilateral vs. bilateral forms, or position of undescended testes, patients with history of cryptorchidism are at higher risk of developing TGCT (15), and suggesting that changes in the spermatogonial stem cell self-renewal and differentiation in cryptorchid testes might be an important pathway leading to TGCT (16).

Taken together, the articles presented in this Research Topic report comprehensive review and original articles dealing with most recent research fields in the pathogenesis, progression, and clinical associations of TGCT.

## Conflict of Interest Statement

The authors declare that the research was conducted in the absence of any commercial or financial relationships that could be construed as a potential conflict of interest.
